# *Cis*-Cotranscription of Two Beta Globin Genes during Chicken Primitive Hematopoiesis

**DOI:** 10.1371/journal.pone.0000703

**Published:** 2007-08-08

**Authors:** Hiroki Nagai, Guojun Sheng

**Affiliations:** Laboratory for Early Embryogenesis, RIKEN Center for Developmental Biology, Kobe, Hyogo, Japan; The Babraham Institute, United Kingdom

## Abstract

Chicken beta globin locus contains four genes, two of which, *rho* and *epsilon*, are expressed from the earliest stage of primitive hematopoiesis. Here we show that the transcription of these two genes in the nucleus engages in “on/off” phases. During each “on” phase, cotranscription of *rho* and *epsilon* in *cis* is favored. We propose that these two chicken beta globin genes are transcribed not by competing for a transcription initiation complex, but in a cooperative way.

## Introduction

**Figure 1:**
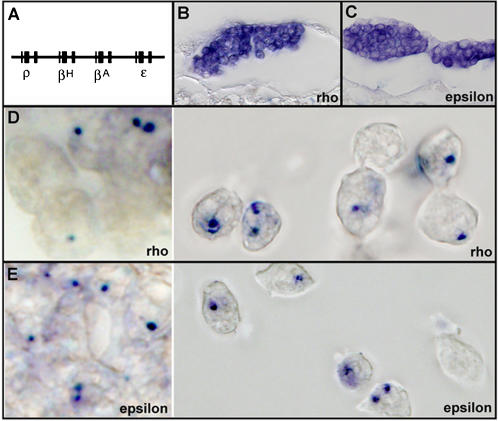
Primary transcript staining of rho and epsilon genes. A) Schematic diagram of chicken beta globin locus. B,C) rho (B) and epsilon (C) cytoplasmic probes stain in all blood cells at stage HH10. D,E) Intron probe against rho (D) or epsilon (E) reveals three types of blood cell staining: negative, single positive and double positive. Left: Sections of HH10 embryo; right: Sections of HH13 embryo.

Studies of beta globin locus have been instrumental in understanding fundamental mechanisms of transcriptional regulation [1], [2]. One unresolved question is whether transcription of two or more beta globin genes can occur simultaneously within a single locus. Chicken beta globin locus, located near the distal tip of chromosome 1, contains four genes (*rho*, *betaH*, *betaA* and *epsilon*) (Fig. 1A) [3]. Chicken embryo establishes its circulation at around stage HH13, and primitive blood differentiation is initiated at HH7 with the expression of *rho* and *epsilon* genes [4]. By HH10, all blood cells are positive for both transcripts in their cytoplasm (Fig. 1B,C).

## Results

To assess the dynamics of primary transcription taking place in the nucleus, we generated probes against intron 2 of *rho* and *epsilon*, and performed whole mount *in situ* with pre-circulation stage embryos. With either *rho* or *epsilon* specific intron probe, we detected three types of primitive blood cells, with their nucleus having 0, 1 or 2 positive signals (Fig. 1D,E). We interpreted them as representing nuclei with both loci off (−/−), one locus on (+/−) and both loci on (+/+), respectively, for a particular globin gene.

A significant percentage of −/− nuclei was seen with either *rho* or *epsilon*, suggesting that chicken beta locus transcription engages in on/off cycles as described in primitive blood cells in mouse. We therefore counted −/−, +/− and +/+ nuclei to determine the relative percentage of each state for stage HH10 embryos. We detected 34.1% of nuclei to be −/−, 50.2% +/− and 15.7% +/+ for *rho* globin (n = 801 nuclei); and 11.7% −/−, 39.0% +/− and 49.2% +/+ for *epsilon* globin (n = 894 nuclei) (Table 1). This indicates that for each locus there is approximately 40% probability to transcribe *rho* and 70% to transcribe *epsilon*.

**Table 1:** Percentages of nuclei with −/−, +/− or +/+ staining for *rho* and *epsilon* single intron staining at HH10.

globin	Total (nuclei)	−/− n (%)	+/− n (%)	+/+ n (%)	Pon (%/locus)	Poff (%/locus)
**rho**	801	273 (34.1)	402 (50.2)	126 (15.7)	39.6	58.4
**epsilon**	894	105 (11.7)	349 (39.0)	440 (49.2)	70.1	34.2

**Figure 2:**
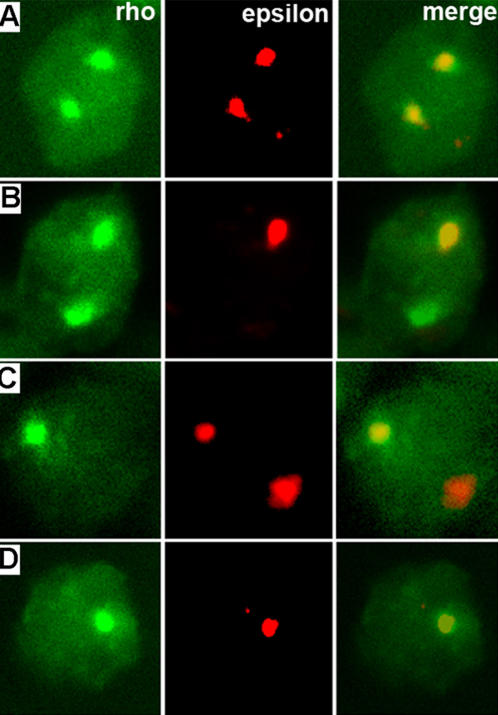
Double staining with rho (green) and epsilon (red) probes reveal significant colocalization (merge). A: ++/++; B: ++/+−; C: ++/−+; D: ++/−−.

**Figure 3:**
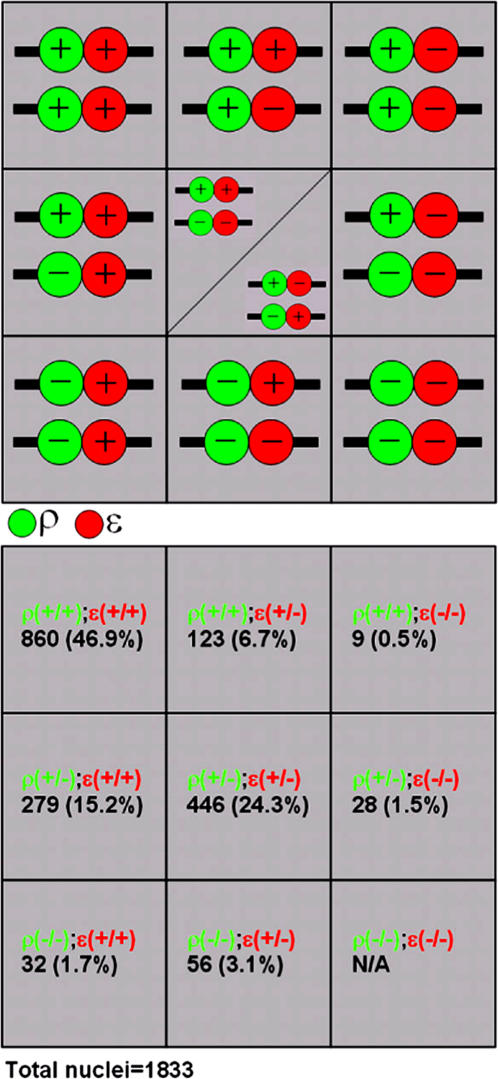
Schematic diagram of 10 possible combinations for rho and epsilon expression and their relative percentages. rho(−/−)epsilon(−/−) is not counted. rho(+/−)epsilon(+/−) data includes both scenarios. The statistics on ++/−− and +−/−+ in the text includes data from this and additional analysis focusing only on rho(+/−)epsilon(+/−) nuclei.

From these ratios we inferred that both beta globin loci in a given nucleus are making similar stochastic choice to be either on or off, because otherwise we would expect skewed ratios among these three states. This led to an obvious question regarding the relationship between *rho* and *epsilon* transcription within a single locus. We used double fluorescent *in situ* to try to discern three possible scenarios: mutually exclusive, cooperative and random. Majority of cells were either *rho* ^(+/+)^ *epsilon* ^(+/+)^, *rho* ^(+/−)^ *epsilon* ^(+/+)^ or *rho* ^(+/+)^ *epsilon* ^(+/−)^ (Figs. 2A-C; 3), for embryos of all stages (HH10-13) examined. In total, these three co-expression combinations represented approximately 70% of all nuclei scored (n = 1833). This would rule out the “mutually exclusive” scenario because it predicts an under-representation of individual loci double positive for both transcripts. A similar observation in mouse, however, was interpreted as a consequence of primary transcripts' half life being comparable to on/off cycle time [5]–[7]. We therefore took advantage of relatively high percentages of *rho* ^(+/−)^ and *epsilon* ^(+/−)^ nuclei seen in single probe *in situ*. We reasoned that if we focus on *rho* ^(+/−)^ *epsilon* ^(+/−)^ nuclei, regardless of primary transcripts' half life, a mutually exclusive scenario would predict +−/−+>>++/−−; a cooperative scenario ++/−−>>+−/−+; and a random scenario ++/−− = +−/−+. We found that over 99% of *rho* ^(+/−)^epsilon^(+/−)^ nuclei were ++/−− (750/753 nuclei) (Fig. 2D). This indicates that not only cotranscription in *cis* of *rho* and *epsilon* can occur, but cooperative cotranscription is preferred. We scored percentages of different *rho* and *epsilon* expression combinations using individual nucleus as a unit, because this allowed us to distinguish ++/−− from +−/−+ scenario. A re-analysis of our data using individual locus as a unit yielded a similar conclusion. Among all loci with at least one signal for either *rho* or *epsilon* (n = 3446), we found 2872 (83.3%) to be *rho* ^+^ *epsilon* ^+^, 402 (11.7%) *rho* ^−^ *epsilon* ^+^ and 172 (5.0%) *rho* ^+^ *epsilon* ^−^.

Our nucleus-based fluorescent *in situ* signal counting, however, yielded higher ratio between +/+ and +/− nuclei for both *rho* and *epsilon* (1.32 and 1.87, respectively) than observed with stage HH10 embryos (0.31 and 1.26, respectively). Since for this analysis we used primarily older stage pre-circulation embryos when individual blood cells are more readily distinguishable, the difference suggested that the probability for each locus to transcribe either *rho* or *epsilon* increases as blood cells mature. Because the increase for *rho* ^(+/+)^/*rho* ^(+/−)^ was relatively large, we examined *rho* single probe stained blood cells in sections with stage HH11 embryos. We observed 23.6% (220/934) +/+, 53.4% (499/934) +/− and 23.0% (215/934) −/− nuclei, indicating an increase of P_on/locus_ for *rho* from 40% at HH10 to 49% at HH11. This rapid increase suggests that immediately after the establishment of circulation after HH13, A vast majority of loci will be transcribing both *rho* and *epsilon*, two predominant beta globins in primitive blood cells.

## Discussion

In summary, our data argue against the mutually exclusive model proposed for mouse primitive erythrocytes [5]–[7]. The only way to explain our data with this model, also called flip-flop or alternating model, is to modify it by adding a two step on/off component. In this “two-step” model, the first on/off decision would be permissive. An off state would have no transcription, while an on state would allow transcription to take place. The second on/off decision would follow a rapid alternating initiation, possible by competing for LCR, of either *rho* or *epsilon*. If this is the case, however, a reinterpretation of mouse data would undermine the basis for the model in the first place. A simpler interpretation of our data is that, at least in chicken, *rho* and *epsilon* are not competing for a single transcription initiation promoting complex. Successful initiation of one gene rather greatly increases the probability of the other to be transcribed.

## Materials and Methods

Fertile chicken eggs were purchased from Shiroyama Farm (Kanagawa, Japan) and incubated at 38.5 degrees to desired stages. Intron probes for *rho* and *epsilon* correspond to exact intron 2 sequences (NCBI #L17432). *In situ* hybridization followed previous described protocol [4] with the only modification that embryos were fixed following incubation within 2 minutes. Single color staining used alkaline phosphatase coupled antibodies, and two color fluorescent staining used probes separately labeled with fluorescein or digoxygenin, revealed stepwise with HRP coupled antibodies using tyramide signal amplification system (PerkinElmer, #NEL753). Counting was performed in 16 um sections for single staining, and in whole mount with isolated blood cells for double staining.
